# BRIVA‐ONE study: 12‐month outcomes of brivaracetam monotherapy in clinical practice

**DOI:** 10.1002/epi4.13078

**Published:** 2024-10-29

**Authors:** Vicente Villanueva, Esther González Villar, Alejandro Fernandez‐Cabrera, Jorge Zurita, Francisco J. Lopez‐Gonzalez, Xiana Rodríguez‐Osorio, Beatriz Parejo‐Carbonell, José C. Estevez, Blanca Mercedes‐Alvarez, Joaquín Ojeda, Marta Rubio‐Roy, Alexandre Garcia‐Escrivá, Asier Gómez‐Ibáñez, Javier Martinez‐Poles, Paula Martinez‐Agredano, Raquel Calle, Alba Sierra‐Marcos, Ana M. Gonzalez, José D. Herrera, Juan Rodriguez‐Uranga, Beatriz Cabezas, Emilio Martinez, Julia Renau, María de Toledo, Kevin G. Hampel, Cristina Alarcón, María Inés Barceló, Angela Monterde, Lidia B. Lara, Gemma Sansa, José M. Serratosa

**Affiliations:** ^1^ Hospital Universitario y Politécnic La Fe. Member of ERN EPICARE Valencia Spain; ^2^ Hospital Universitario y IIS Fundacion Jiménez Diaz Madrid Spain; ^3^ Hospital Universitario Lucus Augusti Lugo Spain; ^4^ Hospital Universitario Infanta Leonor Madrid Spain; ^5^ Complejo Hospitalario Universitario Santiago Santiago de Compostela Spain; ^6^ Hospital Clinico Universitario Madrid Spain; ^7^ Hospital Universitario Reina Sofia Cordoba Spain; ^8^ Hospital Universitario Gregorio Marañón Madrid Spain; ^9^ Hospital Universitario Infanta Sofia Madrid Spain; ^10^ Parc Taulí Hospital Universitari Sabadell Spain; ^11^ Hospital Denia Denia Spain; ^12^ Clinica Universidad de Navarra Madrid Spain; ^13^ Hospital Universitario Rey Juan Carlos Madrid Spain; ^14^ Hospital Universitario Clinico San Cecilio Granada Spain; ^15^ Hospital de la Santa Creu i Sant Pau Barcelona Spain; ^16^ Hospital General La Mancha Centro Alcazar de San Juan Spain; ^17^ Hospital Universitario Virgen de Las Nieves Granada Spain; ^18^ Centro Neurologia Avanzada Sevilla Spain; ^19^ Complejo Asistencial Universitario Leon Leon Spain; ^20^ Hospital Vinaroz Vinaroz Spain; ^21^ Hospital General Universitario Castellon Spain; ^22^ Hospital Universitario La Princesa Madrid Spain; ^23^ Hospital Gomez Ulla Madrid Spain; ^24^ Hospital Universitario Son Espases Palma de Mallorca Spain; ^25^ Hospital Universitari Joan XXIII Tarragona Spain; ^26^ Hospital Parc Tauli Sabadell Spain

**Keywords:** anti‐seizure medication, epilepsy, monotherapy

## Abstract

**Objective:**

This study investigated the effectiveness and tolerability of brivaracetam (BRV) monotherapy in a large series of patients with epilepsy.

**Method:**

This was a multicenter, retrospective, observational, non‐interventional study in 24 hospitals across Spain. Patients aged ≥18 years who started on BRV monotherapy, either as first‐line or following conversion, at least 1 year before database closure were included. Patients were evaluated at baseline and at 3, 6 and 12 months after initiation of BRV monotherapy, in accordance with usual clinical practice at these centers. Data were collected retrospectively from patients' individual charts by participating physicians. The primary effectiveness and safety endpoints were the percentage of seizure‐free patients 1 year after initiation of BRV monotherapy and the proportion of patients reporting adverse events (AEs) over the complete follow‐up period. Retention rates and subpopulation analysis (levetiracetam switchers, elderly and different etiologies) were also investigated.

**Results:**

A total of 276 patients were included (48 with BRV as first‐line monotherapy and 228 who converted to BRV monotherapy). The overall retention rate in monotherapy at 12 months was 89.9% (87.5% for first‐line monotherapy group; 90.4% for conversion‐to‐monotherapy group). Seizure‐freedom rates at 12 months were 77.8% (75% for first‐line monotherapy group; 78.4% for conversion‐to‐monotherapy group). AEs occurred in 39.5% of patients at 12 months (35.4% for first‐line monotherapy group; 40.4% for conversion‐to‐monotherapy group). Most AEs were mild‐to‐moderate. The most frequent AEs were irritability (12.3%) and dizziness (10.1%). The most frequent AEs leading to BRV withdrawal were dizziness (1.8%) and memory problems (1.4%). Similar outcomes in terms of effectiveness and tolerability of BRV monotherapy were observed in patients switching from levetiracetam, those with different epilepsy etiologies, and elderly patients.

**Significance:**

BRV was effective and well tolerated both as first‐line monotherapy and following conversion to monotherapy in a real‐world setting of patients with epilepsy.

**Plain Language Summary:**

The goal of the medical treatment of epilepsy is to ensure best possible patient quality of life, by maximizing seizure control and minimizing medication toxicity. Brivaracetam (BRV) is a new‐generation epilepsy treatment that is well tolerated by patients. In our study, monotherapy with BRV reduced seizures in patients who had not received other treatments and in patients who switched from a previous treatment to BRV monotherapy. BRV was well tolerated and also effective in sensitive patients (i.e., the elderly and those who had epilepsy caused by a brain tumor or a brain injury).

**Key points**

- In patients with epilepsy, brivaracetam was effective and well tolerated as first‐line monotherapy and following conversion to monotherapy.
- The overall retention rate for brivaracetam monotherapy at 12 months was high (89.9%).
- Seizure‐freedom rates at 12 months were 75% for first‐line monotherapy and 78.4% after conversion to monotherapy.
- Brivaracetam monotherapy was well tolerated, with 39.5% of patients reporting adverse events at 12 months.
- Effectiveness and tolerability were similar in patients switching from levetiracetam, those with different etiologies, and in the elderly.

## 1 INTRODUCTION

Epilepsy is one of the most common chronic brain disorders, with a prevalence of approximately 50 million cases worldwide, and affects individuals of all ages.^1^ The armamentarium for medical treatment of epilepsy has increased in the last 30 years with the aim of controlling seizures as soon as possible after diagnosis.^2^ Monotherapy is regarded as both the starting point for medical treatment and the preferred approach (where possible) for patients who have achieved seizure control. In this regard, an ideal monotherapy should provide sufficient antiseizure activity to leave patients seizure free while minimizing adverse effects, leading to an improved quality of life for patients.^3^ This is particularly important for the most vulnerable populations, such as elderly patients in whom comorbidities, polypharmacy and age‐related pharmacokinetics present significant challenges.^4^ The availability of new antiseizure medications (ASMs) is key in achieving these goals.^5^

Brivaracetam (BRV), an analogue of levetiracetam (LEV), is a second‐generation racetam ASM that has been used in the treatment of patients with focal‐onset seizures (FOS).^6^ BRV binds selectively to synaptic vesicle glycoprotein 2A (SV2A) in the brain with a 15–30‐fold higher affinity than LEV, and can therefore be used at lower doses than LEV.^7^, ^8^ In preclinical studies, BRV has demonstrated a higher blood brain barrier permeability compared with LEV, which can be attributed to its higher lipophilicity.^9^ This translates to a 3‐fold faster brain penetration for BRV compared with LEV based on positron emission tomography studies in healthy volunteers.^10^ BRV also has a shorter T_max_ (1 h) compared with LEV (1.3–5.2 h).^11^ Therefore, BRV may be considered a next‐generation LEV with similar effectiveness, faster onset of action and fewer behavioral side effects.

BRV is approved by the Food and Drug Administration (FDA) as monotherapy and adjunctive therapy in patients with FOS aged 1 month and older, and by the European Medicines Agency (EMA) as adjunctive therapy in patients aged 2 years and older. As adjunctive therapy, BRV is well tolerated and has demonstrated significant reductions in seizure frequency compared with placebo in patients with uncontrolled FOS.^12^ Conversion to BRV monotherapy in adults with uncontrolled FOS has been evaluated in two double‐blind, randomized controlled Phase 3 trials using historical controls as a comparator; however, while BRV monotherapy was well tolerated, patient numbers were too low to evaluate the efficacy of BRV in this setting.^6^ While FDA regulatory requirements allowed approval of BRV as monotherapy following extrapolation of adjunctive data, the EMA requires a non‐inferiority study for approval of BRV as monotherapy.

Despite the evolution of drug trials following changes in regulatory requirements,^13^ there remains a lack of monotherapy data for the most recently approved ASMs, including BRV. Real‐world studies present an opportunity to provide monotherapy data for new ASMs outside the confines of randomized controlled trials (RCTs), including in patients who would have been excluded from RCTs due to comorbidities.^14^ However, current data on monotherapy in the real‐world setting are restricted to a small number of patients with limited follow‐up, and most of these patients are included in other series of adjunctive therapy.^15^, ^16^, ^17^, ^18^, ^19^ There is therefore a need for further information regarding BRV monotherapy that could support clinicians in their practice, particularly in settings where no clinical trial data are available, as has been reported with other ASMs.^20^, ^21^, ^22^, ^23^

The aim of this study was to determine the 1‐year effectiveness and tolerability of BRV as monotherapy, both in the first line and following conversion, in a large series of patients with FOS within clinical practice in Spain.

## 2 MATERIALS AND METHODS

### 2.1 Study design

BRIVA‐ONE was a multicenter, retrospective, observational, non‐interventional study to assess the safety and effectiveness of BRV as monotherapy in a series of patients within a real‐world setting. The study protocol was approved by the ethics committee at the Hospital Universitario y Politécnico La Fe and followed the code of ethics set out in the Declaration of Helsinki. The study is reported according to applicable STROBE (STrengthening the Reporting of OBservational studies in Epidemiology) guidelines.^24^

### 2.2 Study participants

Patients were recruited from 24 hospitals with epilepsy or neurology clinics across Spain. Inclusion criteria were: (1) age ≥ 18 years; (2) consecutive patients started on BRV monotherapy, either as first‐line monotherapy or following conversion, at least 1 year before database closure (December 2023); (3) written informed consent by the patient or legal representative according to the study protocol. Exclusion criteria included unreliable information collected in clinical records according to participating clinicians.

### 2.3 Data collection

Patients were evaluated at baseline and at 3, 6 and 12 months after initiation of BRV, in accordance with usual clinical practice at participating centers. Data were collected retrospectively from patients' individual charts by participating physicians. Baseline information included demographic data, seizure type (using 2017 ILAE terminology), etiology, age at epilepsy onset, previous ASMs (if conversion to monotherapy), psychiatric comorbidities, and presence of learning disability. Mean seizure frequency was calculated at baseline (mean monthly seizure frequency during the previous 3 months or, if no seizures, over the previous year), and at each visit (mean monthly seizure frequency since the prior visit). Information regarding the number of seizures was collected from patients' seizure diaries and transcribed to clinical charts. Additionally, physicians reported response at each visit (seizure free, ≥50% responders, no response, or worsening). Adverse events (AEs) considered to be related to BRV by participating physicians were collected at each visit and were graded as mild, moderate or severe. All patients had at least one blood test over the minimum one‐year follow‐up period and vital signs were tested when considered necessary by physicians.

### 2.4 Data analysis and endpoints

The Full Analysis Set (FAS) included all patients who fulfilled the eligibility criteria, started BRV monotherapy treatment, and had at least one efficacy measurement taken after initiation of BRV. The Safety Set included all patients who had received at least one dose of BRV and fulfilled the eligibility criteria. The analysis was performed using the last observation carried forward (LOCF) procedure for missing data. Data are reported for the total population and two subpopulations: (1) patients receiving first‐line treatment with BRV monotherapy (i.e., were not taking other ASMs at the time of BRV initiation); and (2) patients converting to BRV monotherapy (i.e., patients who were previously taking 1 or more other ASMs and were switched to BRV monotherapy). Patients who initiated a second ASM during the observation period were considered to have discontinued BRV monotherapy.

The primary effectiveness endpoint was the percentage of seizure‐free patients at 1 year after initiation of BRV (defined as free from seizures at 1 year and since the 6‐month visit). Secondary effectiveness endpoints included retention‐rate on BRV monotherapy at 1 year, 50% responder rate at 1 year, and the proportion of patients with seizure worsening (i.e., any increase from baseline seizure frequency) at 1 year.

The primary safety endpoint was the proportion of patients reporting one or more AEs over the complete follow‐up period. Secondary safety endpoints included the severity of AEs and the rate of discontinuation due to AEs over the complete follow‐up period.

Exploratory analyses included effectiveness and safety outcomes in specific populations of interest, including patients switching from LEV to BRV, patients with different etiologies (i.e., epilepsy related to brain tumors or traumatic brain injury [TBI]), and patients aged ≥65 years.

### 2.5 Statistical analysis

Analyses were exploratory in nature and there was no imputation for missing values, except in cases of discontinuation where the LOCF procedure was used for the treatment of missing response data. Safety variables were assessed based on the Safety Set and effectiveness variables were assessed using the FAS. Additional predefined subanalyses were performed based on patients who received BRV as first‐line monotherapy compared with those converting to monotherapy, patients switching from LEV to BRV, age ≥ 65 years, and main etiologies at onset of treatment. Seizure freedom rates were calculated on the FAS. The chi‐square test (or Fischer's test, if necessary) was used to compare qualitative variables. Student's t‐test (Mann–Whitney test for non‐normal distributions) was used to compare independent quantitative variables; related quantitative variables were analyzed using the Wilcoxon test. Time‐to‐event variables were assessed using the Kaplan–Meier method. Statistical analyses were conducted using IBM SPSS Statistics 28.0 (IBM Corporation, Armonk, NY, USA). The threshold for statistical significance was 5% (p < 0.05).

## 3 RESULTS

### 3.1 Patient demographics

Data were collected for 276 patients, including 48 patients with BRV as first‐line monotherapy and 228 patients who converted to BRV monotherapy. Reasons for initiating BRV in the conversion‐to‐monotherapy group included lack of effectiveness of other ASMs (68/228 patients [29.8%]), AEs with other ASMs (117/228 patients [51.3%]), both lack of efficacy and AEs with other ASMs (36/228 patients [15.8%]), and other (7/228 patients [3.1%]). Patient demographics and disease characteristics at baseline are shown in Table 1 and Table S1.

**TABLE 1:** Patient demographics and disease characteristics at baseline.

Characteristics	First‐line monotherapy (*n* = 48)	Conversion to monotherapy (*n* = 228)	Overall (*N* = 276)
Female, *n* (%)	29 (60.4)	128 (56.1)	157 (56.9)
Mean age at baseline, years (range)	62.4 (18–92)	52.1 (18–91)	53.9 (18–92)
Mean age at epilepsy onset, years (range)	59.5 (6.7–90.6)	43.1 (0–90)	46 (0–90.6)
Mean baseline seizure frequency, month (SD) [median]	2.12 (5.1) [0.50]	1.44 (3.5) [0.33]	1.63 (4.0) [0.33]
Epilepsy etiology, *n* (%)			
Genetic	1 (2.1)	19 (8.3)	20 (7.2)
Metabolic	0 (0)	1 (0.4)	1 (0.4)
Immunological	0 (0)	2 (0.9)	2 (0.7)
Unknown	25 (52.1)	89 (39)	114 (41.3)
Structural	22 (45.8)	117 (51.3)	139 (50.4)
Tumoral	1 (2.1)	17 (7,5)	18 (6.5)
TBI	0 (0)	13 (5.7)	13 (4.7)
Cortical developmental malformation	2 (4.2)	6 (2.6)	8 (2.9)
Perinatal anoxia	0 (0)	7 (3.1)	7 (2.5)
Cavernoma	1 (2.1)	5 (2.2)	6 (2.2)
Mesial temporal sclerosis	1 (2.1)	5 (2.2)	6 (2.2)
Tuberous sclerosis	0 (0)	4 (1.4)	4 (1.4)
Vascular	0 (0)	3 (1.3)	3 (1.1)
Other	17 (35.4)	53 (23.2)	70 (25.4)
Unclassified	0 (0)	4 (1.8)	4 (1.4)
Mean number of previous ASMs (SD) [range]	0.1 (0.9) [0–6]*	1.9 (1.5) [1–13]	1.6 (1.5) [0–13]
Number of previous ASMs, *n* (%)			
0	47 (98)	0	47 (17.0)
1	1 (2)	134 (58.8)	135 (48.6)
2	0	38 (16.7)	38 (13.8)
3	0	32 (14)	32 (11.6)
≥4	0	24 (10.5)	24 (8.7)

Abbreviations: ASMs, antiseizure medications; SD, standard deviation; TBI, traumatic brain injury.

* One patient had tried 6 ASMs in the past and medication was discontinued after being seizure free for 2 years. BRV was started after seizures reappeared.

### 3.2 Dosage and titration

In all groups, the median BRV dosage was 50 mg on Day 1 and 100 mg at 3, 6 and 12 months. Overall, the mean (range) BRV dosage was 81.5 mg (12.5–300) on Day 1, 116.8 mg (25–300) at 3 months, 121.7 mg (25–400) at 6 months, and 121.3 mg (25–400) at 12 months. In the first‐line monotherapy group, the mean (range) BRV dosage was 79.3 mg (25–200) on Day 1, 100 mg (25–200) at 3 months, 95.7 mg (50–200) at 6 months, and 94.6 mg (50–200) at 12 months. In the conversion‐to‐monotherapy group, the mean (range) BRV dosage was 81.9 mg (12.5–300) on Day 1, 120.3 mg (25–300) at 3 months, 127.1 mg (25–400) at 6 months, and 126.7 mg (25–400) at 12 months.

Regarding BRV titration, 128/271 patients (47.2%) reached the target dosage on the first day (25/46 patients [54.3%] in the first‐line monotherapy group and 103/225 [45.8%] in the conversion‐to‐monotherapy). The most frequent BRV titration scheme was an increase of 25 mg/week (69/271 patients; 25.5%).

### 3.3 Retention

**FIGURE 1:**
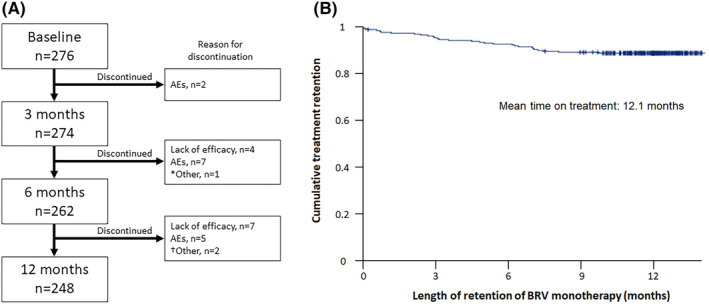
(A) Patient disposition. (B) Treatment retention time (Kaplan–Meier analysis). AEs, adverse events; BRV, brivaracetam. *Patient had TMP prescribed for impulse control by Psychiatry; ^†^One patient had treatment changed at another center for an unknown cause and one patient had treatment changed to lacosamide in the emergency department due to a seizure associated with alcohol intake.

Patient disposition is shown in Figure 1A. Overall BRV monotherapy retention rates were 99.3%, 94.9%, and 89.9% at 3, 6 and 12 months, respectively. Corresponding retention rates on BRV monotherapy were 97.9%, 91.7% and 87.5% in the first‐line monotherapy group and 99.6%, 95.6%, and 90.4% in the conversion‐to‐monotherapy group. The median retention time on BRV monotherapy treatment was 13 months (95% CI 12.6–13.4; Figure 1B). BRV was discontinued in 5 patients (10.4%) in the first‐line monotherapy group, in 12 patients (5.3%) in the conversion‐to‐monotherapy group, and in 17 patients (6.2%) in the whole group.

### 3.4 Effectiveness

**FIGURE 2:**
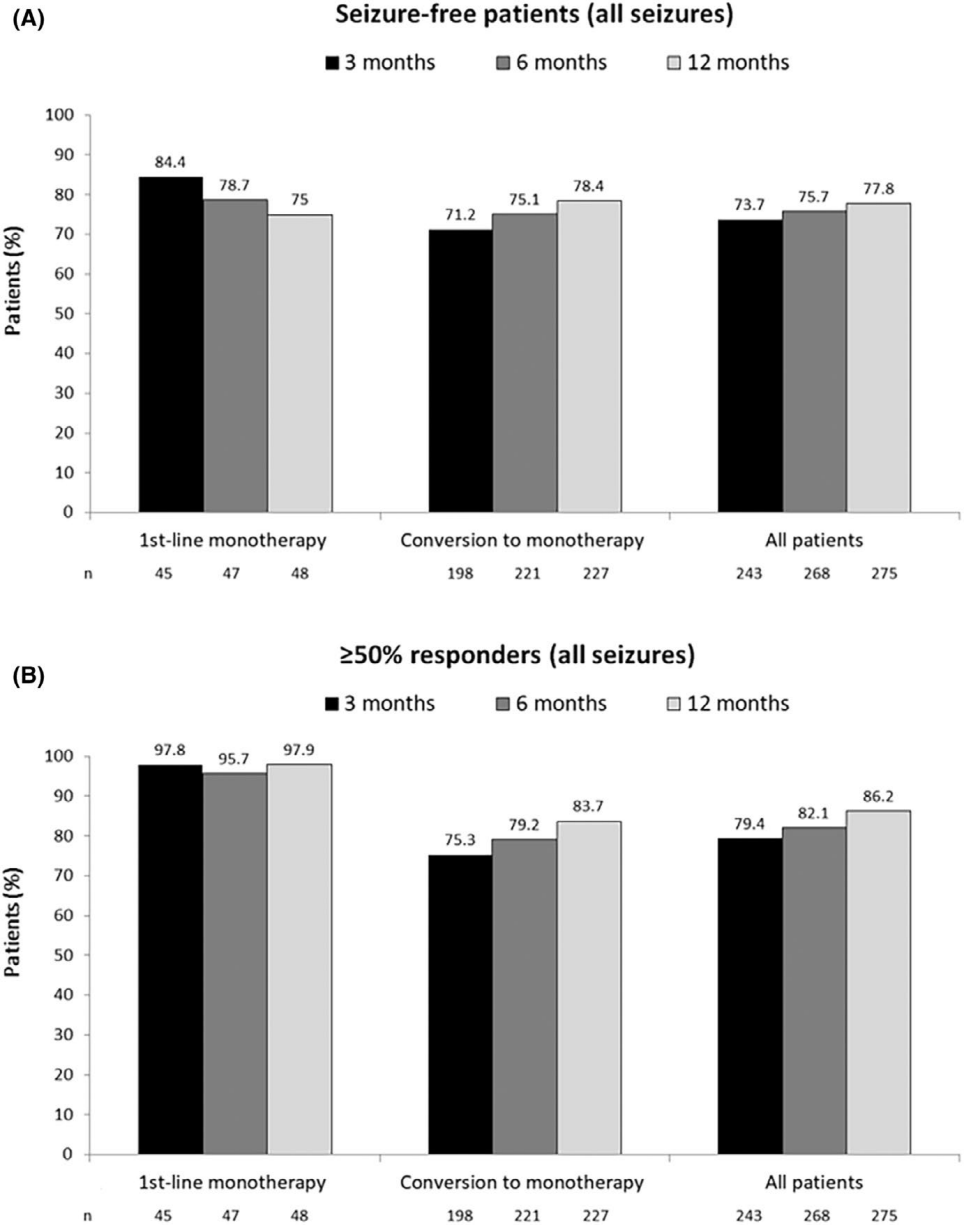
Seizure freedom (A) and ≥ 50% responder rates (B) following first‐line BRV monotherapy or conversion to BRV monotherapy in patients with any type of seizure.

In the FAS, 214/275 patients (77.8%) achieved freedom from all seizures at 12 months, including 75% of patients receiving first‐line BRV monotherapy and 78.4% of patients converted to BRV monotherapy from other ASMs (Figure 2A). Of the 214 patients with seizure freedom at the last visit, 73 (34.1%) had no seizures at baseline. The proportion of patients with ≥50% reduction in the frequency of all seizures (i.e., ≥50% responders) was 86.2% overall, including 97.9% of patients receiving first‐line BRV monotherapy and 83.7% of patients converted to BRV monotherapy from other ASMs (Figure 2B). Worsening of seizure frequency was reported in 13.6%, 11.6% and 9.8% of patients at 3, 6 and 12 months, respectively. Worsening was reported in 2.1% of patients in the first‐line monotherapy at 12 months and in 16.7%, 14% and 11.5% of patients in the conversion‐to‐monotherapy group at 3, 6 and 12 months, respectively.

**FIGURE 3:**
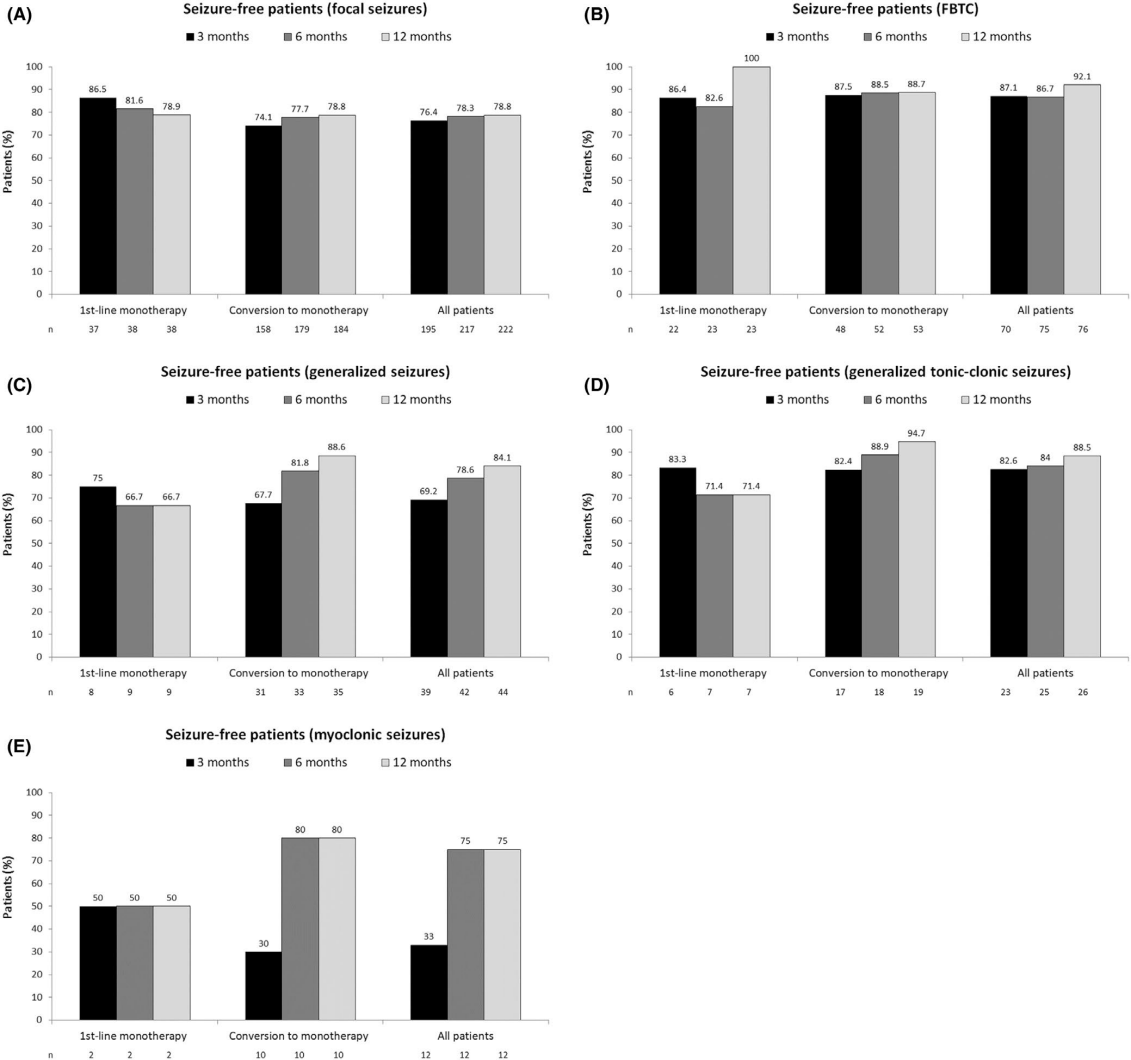
Seizure freedom rates following first‐line BRV monotherapy or conversion to BRV monotherapy in patients with (A) focal onset seizures, (B) focal to bilateral tonic–clonic seizures, (C) generalized onset seizures, (D) generalized onset tonic–clonic seizures, and (E) myoclonic seizures at baseline.

Seizure freedom rates for each seizure type, including focal seizures, FBTCS, generalized onset seizures, GTCS and myoclonic seizures are shown in Figure 3. In patients treated for focal seizures, 69/175 (39.1%) who were seizure‐free at the last visit had no seizures at baseline. In patients with generalized onset seizures, 5/37 (13.5%) seizure‐free patients at the last visit had no seizures at baseline.

### 3.5 Safety

In the Safety Set (*n* = 276), cumulative AEs occurred in 32.4% of patients at 3 months, 36.3% at 6 months, and 39.5% at 12 months (Figure S1). Most AEs were mild‐to‐moderate. The most frequent AEs were irritability (12.3%), dizziness (10.1%), memory disturbances (9.1%), and somnolence (8.7%) (Table 2). No association was found between the presence of psychiatric AEs to BRV and previous psychiatric comorbidity in the complete sample (*p* = 0.077; Chi‐square test).

**TABLE 2:** Adverse events (≥0.5% of patients).

Adverse events, *n* (%)	First‐line monotherapy (*n* = 48)	Conversion to monotherapy (*n* = 228)	Overall (*N* = 276)
Irritability*	5 (10.4)	29 (12.7)	34 (12.3)
Dizziness	5 (10.4)	23 (10.1)	28 (10.1)
Memory disturbances	1 (2.1)	24 (10.5)	25 (9.1)
Somnolence	3 (6.3)	21 (9.2)	24 (8.7)
Fatigue	1 (2.1)	20 (8.8)	21 (7.6)
Depression*	6 (12.5)	13 (5.7)	19 (6.9)
Anxiety*	5 (10.4)	10 (4.4)	15 (5.4)
Ataxia	1 (2.1)	4 (1.8)	5 (1.8)
Laboratory abnormality	3 (6.3)	2 (0.9)	5 (1.8)
Headache	1 (2.1)	3 (1.3)	4 (1.4)
Physical aggressiveness*	1 (2.1)	3 (1.3)	4 (1.4)
Verbal aggressiveness*	2 (4.2)	2 (0.9)	4 (1.4)
Cutaneous	0 (0.0)	2 (0.9)	2 (0.7)
Decreased appetite	2 (4.2)	0 (0.0)	2 (0.7)
Insomnia	1 (2.1)	1 (0.4)	2 (0.7)
Tremor	0 (0.0)	2 (0.9)	2 (0.7)
Adverse events leading to brivaracetam withdrawal, *n* (%)	First‐line monotherapy (*n* = 5)	Conversion to monotherapy (*n* = 9)	Overall (*n* = 14)
Dizziness	1 (2.1)	4 (1.8)	5 (1.8)
Memory problems/bradypsychia	0 (0.0)	4 (1.8)	4 (1.4)
Anxiety*	2 (4.2)	1 (0.4)	3 (1.1)
Irritability*	0 (0)	3 (1.3)	3 (1.1)
Decreased appetite	2 (4.2)	0 (0.0)	2 (0.7)
Depression*	2 (4.2)	0 (0.0)	2 (0.7)
Physical aggressiveness*	1 (2.1)	1 (0.4)	2 (0.7)
Verbal aggressiveness*	1 (2.1)	1 (0.4)	2 (0.7)

*Note*: Anosmia, breast pain, constipation, cramps on legs, decreased libido, erectile dysfunction, hypogeusia, increased appetite, mood changes, nasosinus symptoms, nausea/vomiting, psychosis, and sphincteric incontinence were reported by 1 patient (0.4%) in the conversion‐to‐monotherapy group.

* Psychiatric adverse events.

AEs leading to treatment discontinuation occurred in 14/276 patients (5.1%), including 5/48 (10.4%) in the first‐line monotherapy group and 9/228 (3.9%) in the conversion‐to‐monotherapy group. The most frequent AEs leading to BRV withdrawal were dizziness (5/228; 1.8%), memory problems (4/228; 1.4%), anxiety (3/228; 1.1%), and irritability (3/228; 1.1%) (Table 2). Psychiatric AEs leading to treatment discontinuation occurred in no patients at 3 months, five patients (1.9%) at 6 months, and 9 patients (3.3%) at 12 months (including four in the first‐line monotherapy group and 5 in the conversion‐to‐monotherapy group).

### 3.6 Exploratory analyses

#### 3.6.1 LEV‐to‐BRV switch

During the study, 152 patients taking LEV were switched to BRV monotherapy. Nineteen patients switched because of a lack of efficacy of LEV and 133 patients switched because of AEs (115 because of psychiatric AEs). Baseline characteristics, efficacy and safety outcomes in these patients are detailed in Table 3. Following the switch from LEV to BRV, the proportion of patients who were seizure‐free was 76% (98/129) at 3 months, 77.7% (115/148) at 6 months, and 78.8% (119/151) at 12 months. In total, 115 patients switched from LEV to BRV because of psychiatric AEs (including 15 patients who were switched due to both psychiatric and non‐psychiatric AEs). Of these, 47 patients (40.9%) had a prior psychiatric comorbidity. At 12 months, 22/115 patients (19.1%) reported psychiatric AEs with BRV, of which 8 (36.4%) had a prior psychiatric comorbidity. There was no significant association between psychiatric AEs with BRV and prior psychiatric comorbidity (p = 0.633). Two patients (1.7%) who switched from LEV to BRV because of psychiatric AEs discontinued BRV due to AEs; both patients had at least one psychiatric AE and one patient had a prior psychiatric comorbidity. There was no significant association between discontinuation of BRV in patients with psychiatric AEs and prior psychiatric comorbidity (*p* = 0.791).

**TABLE 3:** Baseline characteristics, efficacy and safety outcomes in patients switched from levetiracetam to brivaracetam.

	All	Switch due to lack of efficacy	Switch due to AEs	Switch due to psychiatric AEs
Switched, *n* (%)	152	19 (12.5)	133 (87.5)^a^	115 (75.7)^b^
Timing of switch^b^, *n* (%)				
Overnight	95 (62.5)			
Progressive transition	57 (37.5)			
Seizure free, *n*/*N* (%)				
3 months	98/129 (76)	5/13 (38.5)	93/116 (80.2)	81/99 (81.8)
6 months	115/148 (77.7)	9/17 (52.9)	106/131 (80.9) 111/132 (84.1)	91/113 (80.5) 97/114 (85.1)
12 months	119/151 (78.8)	8/19 (42.1)		
Seizure free at last visit and no seizures at baseline, *n*/*N* (%)	57/119 (47.9)			
AEs over 12 months, *n*/*N* (%)	55/152 (36.2)	5/19 (26.3)	50/133 (37.6)	42/115^c^ (36.5)
Mild, %	19.1	0	19.5	18.3
Moderate, %	15.1	15.8	17.3	17.4
Severe, %	2	10.5	0.8	0.9
AEs leading to BRV withdrawal, n/N (%)	5/152 (3.3)	1/19 (5.3)	4/133 (3)	3/115 (2.6)
Patients with ≥1 psychiatric AE, n	3	1	2	2^d^

AEs, adverse events; BRV, brivaracetam; LEV, levetiracetam.

^a^ 100 patients reported only psychiatric AEs, 14 patients reported only non‐psychiatric AEs, 15 patients reported psychiatric and non‐psychiatric AEs, and 2 patients did not report the type of AEs.

^b^ Includes 15 patients who were switched due to both psychiatric and non‐psychiatric AEs. For the overnight switch, BRV was initiated the day after stopping LEV using a BRV dosage 1/10th–1/15th of the LEV dosage. The progressive transition (decreasing the dosage of LEV and increasing the dosage of BRV) was carried out over 1–3 weeks. The decision on the switch method to use depended on the physicians' choice as there were no specific recommendations in this regard.

^c^ 22/115 patients (19.1%) reported psychiatric AEs with BRV, of which 8 (36.4%) had a prior psychiatric comorbidity.

^d^ One patient had a prior psychiatric comorbidity; there was no significant association between discontinuation of BRV in patients with psychiatric AEs and prior psychiatric comorbidity (*p* = 0.791).

#### 3.6.2 Elderly patients

In total, 105 patients were aged ≥65 years (mean [range] 76 years [65–92]), with a mean age at epilepsy onset of 70.6 years (11.4–90.6). Of these, 47/105 (44.8%) were transitioned from LEV to BRV, including 7/47 (14.9%) because of a lack of efficacy and 40/47 (85.1%) because of AEs. Psychiatric comorbidities were present in 37/104 patients (35.6%) at baseline. The median BRV dosage was 50 mg at day 1 and 100 mg at 3, 6 and 12 months. At 12 months, 87.6% of patients were still on BRV monotherapy. The percentage of seizure‐free patients at 3, 6 and 12 months was 82%, 79.4% and 81.9%. AEs were reported by 43/105 patients (41%; Table S2) and led to BRV withdrawal in 9.5% patients.

#### 3.6.3 Patients assessed by structural etiology

The most frequent structural etiologies were brain‐tumor related epilepsy (n = 18) and epilepsy associated with TBI (*n* = 13). Baseline characteristics and safety outcomes in these patients are detailed in Table S3. The percentage of seizure‐free patients at 3, 6, and 12 months was 75%, 77.8% and 83.3%, respectively, in those with brain‐tumor related epilepsy and 91.7%, 92.3% and 92.3% in those with TBI‐related epilepsy.

## 4 DISCUSSION

In this real‐world analysis of a large series of patients with a long follow‐up period, the effectiveness and safety of BRV monotherapy was demonstrated. Seizure‐freedom rates up to 1 year were consistently above 70% in the entire BRV monotherapy population and were at least 75% in patients receiving BRV monotherapy as first‐line treatment. In addition, AEs were typically mild or moderate. Better outcomes observed with first‐line monotherapy compared with conversion‐to‐monotherapy could be related to the higher mean age at epilepsy onset in this group, which is typically associated with better responses, and to the lower number of prior ASMs. Moreover, outcomes in this cohort should be interpreted cautiously as patients had low seizure frequency at the time of inclusion and around a third were seizure free at initiation of the study.

The lack of available data for BRV monotherapy complicates comparisons. A retrospective real‐world study in 44 patients converted to BRV monotherapy reported slightly lower seizure‐freedom rates (72.7% at 6 months; 58.1% at 12 months) than those seen in our study.^15^ In line with the high response rate observed in our study, a recent study in 114 patients with FBTCS or primary GTCS reported rates of seizure freedom from FBTCS/GTCS of 89.7% at 6 months in the 35 patients who started BRV as monotherapy.^17^ Similarly, a large, retrospective, observational study in 615 patients with epilepsy claimed a seizure‐freedom rate of 58% at 3 months in a subset of 19 patients receiving BRV monotherapy.^18^ Only the results from the EXPERIENCE study, an international pooled analysis of 1644 individual patient records, observed lower seizure‐freedom rates in patients receiving BRV monotherapy (58.1%, 34.5% and 36% at 3, 6 and 12 months, respectively) than those observed in our study.^16^ Moreover, our study is consistent with sustained efficacy of BRV over time, as supported by prior publications.^25^, ^26^

Caution should be applied when comparing seizure‐freedom rates for BRV monotherapy across real‐world studies for several reasons: most series include low numbers of patients; patients' baseline characteristics may differ between studies; and most studies do not clarify if patients received BRV as first‐line monotherapy or following conversion to monotherapy. Nevertheless, the majority of studies report seizure‐freedom rates of over 50% with BRV monotherapy, similar to those observed with other ASMs in a comparable setting (e.g., 12‐month seizure‐freedom rates of 60.2% for lacosamide, 56.6% for eslicarbazepine acetate, 50.5%–58.7% for LEV, 64.5% for valproate and 56.7% for carbamazepine as first‐line monotherapy).^21^, ^22^, ^23^

With regard to different seizure types, the percentage of patients who were free of FBTCS after BRV monotherapy in our study (87.1%, 86.7% and 92.1% at 3, 6 and 12 months) was slightly higher than outcomes reported by Fonseca et al., including 114 patients with FBTCS and GTCS (69.1%, 68.3% and 73.4% at 3, 6 and 12 months).^17^ In a series of 37 patients who received BRV (12 as monotherapy) for generalized onset seizures, 62.2% were seizure‐free at 6 months.^27^ This compares with 6‐month data from our study in which 78.6% of patients were free of generalized onset seizures, 75% were free of GTCS, and 84% were free of myoclonic seizures. These differences may be because our study included only monotherapy patients whereas the other studies also included patients receiving adjunctive therapy, which is usually associated with a worse outcome. From a clinical perspective, efficacy outcomes with BRV monotherapy appear to be in line with other ASMs and support its use in clinical practice. The effectiveness of BRV monotherapy in reducing seizures of different types also suggests its usefulness in patients in whom seizure classification is unclear.

The predominant AEs observed in our study (i.e., irritability, dizziness, somnolence and memory disturbances) were similar to those previously reported in adjunctive therapy studies of BRV.^28^ Overall, we observed higher cumulative AE rates (36.3% at 6 months; 39.5% at 12 months) than reported by Lattanzi et al. (13.6% at 6 months)^15^ and in the EXPERIENCE study (3.8% at 12 months).^16^ However, the proportion of patients with AEs leading to treatment discontinuation was similar in our study (2.3% at 6 months and 3.9% at 12 months in the conversion‐to‐monotherapy group) and the study by Lattanzi et al. (4.5% at 6 months).^15^ The retention rate in our study (89.9% at 1 year) was similar to that reported by Lattanzi et al. (83.9% at 12 months).^15^ When comparing with other ASMs, AE rates with first‐line BRV monotherapy were in line with those reported for lamotrigine in the SANAD II trial in patients with newly diagnosed focal epilepsy, but lower than rates for LEV or zonisamide.^29^ These outcomes support the good tolerability profile of BRV monotherapy, which is especially important from a clinical perspective as monotherapy patients typically have better seizure control and fewer AEs than refractory patients so may be less willing to tolerate AEs with a new ASM.

The adequate dosage for BRV monotherapy is uncertain due to a lack of data. The median BRV dosage in our study (50 mg/day on Day 1; 100 mg/day at other timepoints) was slightly lower than that used in the Italian monotherapy series (150 mg/day at 6 months and 125 mg/day at 12 months),^15^ but similar to that used in the monotherapy group of the EXPERIENCE study (100 mg/day on Day 1 and at 12 months). In elderly patients, the median BRV dosage was lower at 12 months (100 mg/day) compared with the Italian adjunctive therapy series (150 mg/day).^30^ Consequently, it seems that 100 mg/day can be considered as the usual maintenance dosage in monotherapy. A key target in BRV dosing is to achieve a therapeutic dosage by Day 1 as this has been shown to improve early efficacy outcomes, including rates of seizure‐freedom at 3 and 6 months.^17^, ^28^ In our study, approximately half of the patients (47.2%) reached the target BRV dosage on Day 1. This has particular clinical significance in the monotherapy setting, where seizure control is dependent on a single ASM.

As BRV has an acceptable safety profile and few known drug–drug interactions, we explored its effectiveness and safety in patients with epilepsy etiologies frequently underrepresented in clinical trials because of comorbidities and patient fragility. In both patients with brain tumor‐related epilepsy and those with epilepsy related to TBI, we observed a higher rate of seizure freedom at 12 months compared with the overall population. In the EXPERIENCE study, patients with brain tumor‐related epilepsy also had a slightly higher seizure‐freedom rate than patients with a different etiology (18.2% vs. 15.8%); similarly, the rate of seizure freedom at 12 months in patients with epilepsy related to TBI was higher compared with other etiologies (17.2% vs. 15.9%), although much lower than in our study (17.2% vs. 92.3%).^31^ In an Italian series, patients with brain tumor‐related epilepsy receiving add‐on BRV to ASM treatment had a significant reduction in mean monthly seizure frequency, with 60.6% of patients achieving seizure‐freedom.^32^ Comparisons between our study and those previously published is complicated by our inclusion of monotherapy patients, a population considered easier to treat than the more refractory populations included in other studies. However, there was still a tendency towards better outcomes in patients with epilepsy related to brain tumors or TBI compared with the overall population. Together, these findings suggest the usefulness of BRV monotherapy in epilepsy etiologies characterized by significant comorbidities and patient fragility.

While there is uncertainty regarding relative outcomes between different ASMs in the elderly due to a lack of studies, particularly in monotherapy,^33^ we found comparable outcomes in BRV‐treated elderly patients and the overall population; these included similar rates for retention (87.6% vs. 89.9%), seizure freedom (81.9% vs. 77.8%) and AEs (41% vs. 39.5%) at 12 months. An Italian series, including 111 patients aged at least 65 years receiving BRV as adjunctive treatment (and consequently more refractory than our series), reported a lower seizure freedom rate of 31.5% at 1 year and a lower AE rate of 24.2% compared with our study;^30^ the most common AEs were somnolence, vertigo, nervousness/agitation and fatigue, in line with AEs reported in the current work. We consider this information particularly relevant from a clinical point of view as many patients with late‐onset epilepsy can be controlled on monotherapy.

Although switching from LEV to BRV has previously been explored, there is scarce monotherapy data in this setting. In our study, we observed a 78.8% seizure freedom rate at 12 months in patients who switched from LEV to BRV due to any reason and a 42.1% rate in those patients who switched due to a lack of efficacy with LEV. These results are similar to those of the retrospective study by Snoeren et al. which found that 46.2% of LEV non‐responders responded to BRV,^34^ and adds to growing evidence that treatment failure with LEV should not preclude the use of BRV, whether LEV is discontinued due to lack of efficacy or not.^35^

Regarding AEs, we found no association between psychiatric AEs with BRV and prior psychiatric comorbidities. Several studies have reported reductions in behavioral AEs with BRV compared with LEV.^36^, ^37^, ^38^ In our study, patients who switched from LEV to BRV due to psychiatric AEs had a psychiatric AE rate of only 19.1%.

This study is limited by its observational, nonblinded, uncontrolled design and the data must therefore be interpreted carefully. Baseline seizure status was unknown in 12 patients and we cannot exclude underrepresentation of AEs and discontinuations. The main strength of this study is that it includes a large population of patients receiving BRV monotherapy and followed during a long period in a real‐world setting, which is valuable considering the scarcity of reported data in this setting.

In conclusion, this study supports the effectiveness and safety of BRV monotherapy in patients with epilepsy in a real‐world setting, including patients switching from LEV, those with different epilepsy etiologies, and the elderly. BRV may be considered a next‐generation racetam with some advantages with respect to LEV.

## AUTHOR CONTRIBUTIONS

All authors contributed to the conception and design of the study and to acquisition of data. Vicente Villanueva and Jose Maria Serratosa organized the database, analyzed the data, wrote the manuscript, and created tables and figures. All authors discussed the results, revised the first draft, and contributed to the final manuscript.

## CONFLICT OF INTEREST STATEMENT

VV has received honoraria and/or research funds from Angelini Pharma, Bial, Eisai, Jazz Pharmaceuticals, Neuraxpharm, Novartis, Nutricia, Takeda, UCB Pharma and Xenon. JZ has received consultant and/or speaker honoraria from Alter, Angelini Pharma, Bial, Eisai, Jazz Pharmaceuticals, Neuraxpharm and UCB Pharma. FJLG has received consultant and/or speaker honoraria from Angelini Pharma, Bial, Eisai, Esteve, GW Pharmaceutical Company (now a part of Jazz Pharmaceuticals), LivaNova, Nutricia and UCB Pharma. XRO has received consultant and/or speaker honoraria from Angelini Pharma, Bial, Eisai, Jazz Pharmaceuticals, Livanova, Neuraxpharm and UCB Pharma. BPC has received consultant and/or speaker honoraria from Angelini Pharma, Bial, Eisai, Jazz Pharmaceuticals and UCB Pharma. BMA has received consultant and/or speaker honoraria from Angelini‐Pharma, Bial, Eisai, Kern Pharma, Jazz Pharmaceuticals, Nutricia and UCB Pharma. AGI has received consultant and/or speaker honoraria from Angelini Pharma, Bial, Eisai, Idorsia, Jazz Pharmaceuticals, Neuraxpharm and UCB Pharma. JMP has received speaker honoraria from Eisai and UCB Pharma. PMA has received honoraria and/or research funds from Angelini Pharma, Bial, Eisai, Jazz Pharmaceuticals, Kern Pharma, Livanova, Neuraxpharm and UCB Pharma. RC has received consultant and/or speaker honoraria from Angelini Pharma, Eisai, Neuraxpharm and UCB Pharma. ASM has received consultant and/or speaker honoraria from Angelini‐Pharma, Eisai, Ferrer, GW Pharmaceutical Company (now a part of Jazz Pharmaceuticals), Kern Pharma, Neuraxpharm, Nutricia, Takeda and UCB Pharma. JDH has received consultant and/or speaker honoraria from Angelini Pharma, Bial, Eisai, and UCB Pharma. JRU has received honoraria and/or research funds from Angelini Pharma, Bial, Eisai, Jazz Pharmaceuticals, Neuraxpharm, Novartis, Nutricia, Takeda, UCB Pharma and Xenon. JMS has received honoraria for advisory, educational activities, and/or research funds from Angelini Pharma, BIAL, Eisai Inc., Esteve, Ferrer, Jazz Pharmaceuticals, GW Pharmaceuticals, Sanofi, UCB Pharma, and Zogenix. The remaining authors have no conflicts of interest. We confirm that we have read the Journal's position on issues involved in ethical publication and affirm that this report is consistent with those guidelines.

## Supporting information


Data S1.

## Data Availability

The data that support the findings of this study are available from the corresponding author upon reasonable request.
