# Supplementary material for: BRIVA‐ONE study: 12‐month outcomes of brivaracetam monotherapy in clinical practice
Source: Epilepsia Open. 2024 Oct 29;9(6):2429–42. doi: 10.1002/epi4.13078 (PMC11633701; doi:10.1002/epi4.13078)
Supplement: Supplementary file 1 — Data S1. [file EPI4-9-2429-s001.docx]

**Supporting information**

***Figure 1: Adverse event rate and severity***


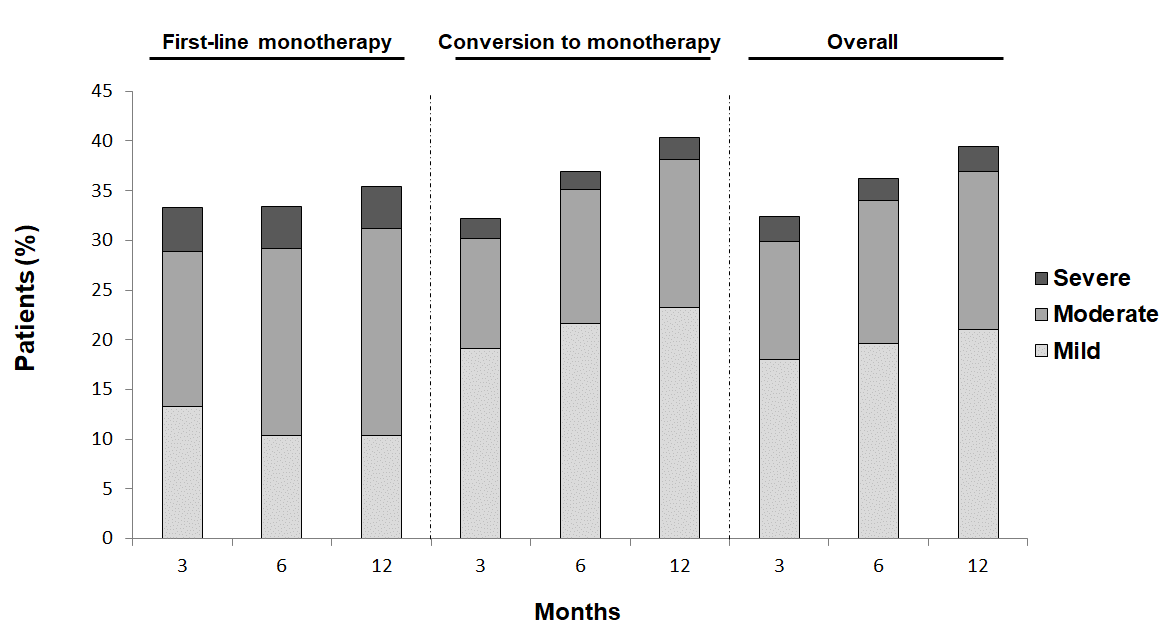


***Table 1. Additional patient demographics and disease characteristics at baseline***

| **Characteristics** | **First-line monotherapy** | **Conversion to monotherapy** | **Overall** |
| --- | --- | --- | --- |
| Prior psychiatric comorbidity, n/N (%)  Depression  Anxiety  Personality disorder Psychosis  Hyperactivity  Other | 18/47 (38.3) | 77/226 (34.1) | 95/273 (34.8)  58/273 (21.2)  42/273 (15.4)  11/273 (4.9)  6/273 (2.2)  7/273 (2.6)  8/273 (2.9) |
| Intellectual disability, n/N (%) | 4/48 (8.3) | 29/224 (12.9) | 33/272 (12.1) |
| Baseline seizures*, n/N (%) | 43/43 (100) | 143/221 (64.7) | 186/264 (70.5) |
| Patients with FOS diagnosis and known baseline seizure status, n/N (%)  FOS at baseline  FBTCS at baseline | 33/34 (97.1)  23/34 (67.6) | 107/179 (59.8)  53/179 (29.6) | 140/213 (65.7)  76/213 (35.7) |
| Patients with generalized onset seizure diagnosis and known baseline seizure status, n/N (%)  Generalized seizures at baseline | 9/9 (100) | 26/33 (78.8) | 35/42 (83.3)^†^ |
| Number of prior ASMs  Mean (SD)  Median (IQR) |  | 1.9 (1.5)  1 (1–2) |  |
| Number of concomitant ASMs at start of BRV treatment, n/N (%)  Mean (SD)  Median (IQR)  1  2  3  4 |  | 1.1 (0.4)  1 (1–1)  210/228 (92.9)  15/228 (6.6)  2/228 (0.9)  1/228 (0.4) |  |
| Most frequent concomitant ASMs, n/N (%)  Levetiracetam  Lacosamide  Lamotrigine  Clobazam  Eslicarbazepine acetate  Valproic acid  Carbamazepine  Oxcarbazepine  Phenytoin |  | 152/228 (66.7)  26/228 (11.4)  13/228 (5.7)  11/228 (4.8)  10/228 (4.4)  8/228 (3.5)  6/228 (2.6)  6/228 (2.6)  5/228 (2.2) |  |

*Patients with known baseline seizure status; for those 12 patients whose seizure status was unknown at baseline, response over the follow-up was reported and therefore the patients were not excluded from the analysis.

^†^Seizure types included GTCS (n=26), myoclonic seizures (n=12) and absence seizures (n=1).

ASMs, antiseizure medications; FBTCS, focal to bilateral tonic-clonic seizures; GTCS, generalized onset tonic-clonic seizures; IQR, interquartile range; SD, standard deviation; TBI, traumatic brain injury.

***Table 2. Adverse events in patients aged ≥65 years***

|  | **N=105** |
| --- | --- |
| AEs, n (%)  Dizziness  Irritability  Memory problems/bradypsychia  Somnolence  Anxiety  Fatigue  Verbal aggressiveness  Ataxia  Depression  Headache  Laboratory abnormality  Physical aggressiveness | 43 (41)  13 (12.4)  11 (10.5)  8 (7.6)  6 (5.7)  5 (4.8)  5 (4.8)  5 (4.8)  4 (3.8)  4 (3.8)  3 (2.9)  3 (2.9)  2 (1.9) |
| AEs leading to BRV withdrawal, n (%) | 10 (9.5) [5 patients had ≥1 psychiatric AE] |

AEs, adverse events; BRV, brivaracetam; LEV, levetiracetam.

***Table 3. Baseline characteristics and safety outcomes in patients according to structural etiology****

|  | **Brain-tumor related (n=18)** | **Traumatic brain injury-related (n=13)** |
| --- | --- | --- |
| Mean age, years (range) | 51.1 (21–83) | 60.2 (34–80) |
| Mean age at epilepsy onset, years (range) | 44.2 (5.9–81.6) | 49.2 (20.9–79.7) |
| Transitioned from LEV to BRV, n/N (%)  Due to AEs | 11/18 (61.1)  10/11 (90.9) | 11/13 (84.6)  11/13 (84.6) |
| Psychiatric comorbidity, n/N (%) | 7/11 (38.9) | 5/13 (38.5) |
| Median BRV dosage, mg  Day 1  3 months  6 months  12 months | 100  100  100  125 | 100  137.5  125  125 |
| Still on BRV monotherapy at 12 months, n/N (%) | 17/18 (94.4) | 13/13 (100) |
| AEs, n/N (%) | 5/18 (27.81) | 4/13 (30.8) |
| AEs leading to BRV withdrawal, n (%) | 1 (the patient had ≥1 psychiatric AE) | 0 |

AEs, adverse events; BRV, brivaracetam; LEV, levetiracetam.

*3 patients had vascular etiology, all of whom switched from LEV to BRV (2 due to lack of efficacy of LEV and 1 due to psychiatric AEs).
